# Whole body effective dose equivalent dataset for MAX and FAX shielded with Common Aerospace Materials in deep space

**DOI:** 10.1016/j.dib.2019.104885

**Published:** 2019-11-26

**Authors:** Daniel Bond, Braden Goddard, Robert Singleterry, Sama Bilbao y León

**Affiliations:** a Department of Mechanical and Nuclear Engineering, Virginia Commonwealth University, 401 West Main Street P.O. Box 843015 Richmond, VA 23284, USA; b NASA Langley Research Center, MS 188E, 2 West Reid Street, Hampton, VA 23681, USA; c Nuclear Energy Agency, 46, Quai Alphonse Le Gallo, 92100 Boulogne-Billancourt, France

**Keywords:** Spacecraft, Materials, OLTARIS, Deep space, MAX, FAX

## Abstract

Materials have a primary purpose in the design of space vehicles, such as fuels, walls, racks, windows, etc. Additionally, each will also affect space radiation protection. Using the On-Line Tool for the Assessment of Radiation in Space (OLTARIS), version 3.5, analysis package, this article includes the whole body effective dose equivalent (E_D_) data from human phantoms being shielded by 59 aerospace materials for deep space travel. To represent the average anatomy of an astronaut, the Female Adult voXel (FAX), 2005 version, and the Male Adult voXel (MAX), 2005 version, human phantoms are used. A simple spherical geometry, which is composed of a spherical shell with the human phantom placed in the center, is also used. Eighteen shielding thicknesses ranging from 0.01 to 1000 g per centimetres squared are evaluated and the ray distribution used in this study is the 1002 geodesic. All aerospace materials are categorized into four groups: metals, polymers, composites, and fuels, hydrides, and liquid gases. These materials include common fuels and propellants used in space travel, engineered materials developed to significantly increase the absorption of secondary radiation, and materials in the early stages of development for the purpose of meeting both shielding and structural needs of future spacecraft missions. The data in this article is used for the paper, “Evaluating the Effectiveness of Common Aerospace Materials at Lowering the Whole Body Effective Dose Equivalent in Deep Space,” [13].

**Table:** Specifications Table

Subject	Aerospace Engineering
Specific subject area	Aerospace material's impact on deep space radiation
Type of data	Tables
How data were acquired	OLTARIS, the On-Line Tool for the Assessment of Radiation in Space, version 3.5.
Data format	Raw
Parameters for data collection	Two deep space boundary conditions were used. For galactic cosmic rays, the 1977 solar minimum, and the October 19–24, 1989 event measured by the GOES 7 spacecraft, for solar particle events.
Description of data collection	Using OLTARIS version 3.5, the Ray-by-Ray method and 1002 geodesic ray distribution were utilized. The thickness distributions analysed were 0.01, 0.1, 0.3, 0.5, 0.75, 1.0, 3.0, 5.0, 7.5, 10, 30, 50, 75, 100, 300, 500, 750 and 1000 g/cm2.
Data source location	NASA Langley Research Center, Hampton, Virginia, USA and Virginia Commonwealth University, Richmond, VA, USA
Data accessibility	With the article
Related research article	D.K. Bond, B. Goddard, R.C. Singleterry, S. Bilbao y León, Evaluating the effectiveness of common aerospace materials at lowering the whole body effective dose equivalent in deep space, Acta Astronautica [12]

**Table:** 

**Value of the Data** • This dataset shows material evaluations that can be used to potentially help design spacecrafts for extended Lunar and Martian missions. • Companies and scientists that are working towards designing spacecrafts to protect astronauts from deep space radiation during extended missions. • This data shows patterns in material effectiveness to lower exposure to astronauts, that can be used in directing material development for shielding purposes in deep space missions.

## 1 Data

The data compiled in this article is the whole body effective dose equivalent (E_D_) absorbed within the male adult voxel (MAX) [1] and female adult voxel (FAX) [2]human phantom calculated using the On-Line Tool for the Assessment of Radiation in Space (OLTARIS) [[3], [4], [5]], version 3.5. The 59 shielding materials (Table 1) evaluated are separated into four categories: metals, polymers, composites and fuels, hydrides and liquid gases.

**Table 1:** Material names separated into four categories metals, polymers, composites, and fuels, hydrides and liquid gases.

Metals	Fuels, Hydrides & Liquid Gases	Polymers	Composites
Aluminum	Methane	Polyethylene	Graphite Epoxy (51/49)
310 Stainless Steel	Liquid Hydrogen	Acrylic	IM7/977-3 Graphite Epoxy
Aluminium Alloy 2195	Liquid Oxygen	Water	Borated Polyethylene (Natural)
Lead	Monomethyl hydrazine	Epoxy	Borated Polyetherimide (Natural)
Martian Regolith	Kerosene (n-Dodecane)	Polyetherimide/Ultem 1000	Boron Nitride Nanotubes (^10^B)
Lunar Regolith	Kerosene (RG-1/RP-1)	Polyimide	Borated Polyethylene Epoxy - Natural
Silicon Dioxide (Silica)	Solid Propellant	Polysulfone	Lunar Regolith - 20% Epoxy
Tantalum	Beryllium Hydride	Polytetrafluoroethylene/Teflon	Carbon Nanotubes w/6.5% H
Silicon	Magnesium Hydride	Noryl 731	Boron Nitride Nanotubes w/7.14% H
Tungsten	Lithium Hydride (^6^Li)	Tissue	Martian Regolith w/20% Epoxy
Titanium	Lithium Hydride (Natural)	Carbon Nanotube	Boron Nitride Nanotubes (Natural)
Copper	Sodium Borohydride	Polyethylene Epoxy	Carbon Nanotubes w/20% H
	Magnesium Borohydride		Polyethylene Epoxy with Carbon
			Boron Nitride Nanotubes w/20% H
			Borated Polyethylene (^10^B)
			Carbon Nanotubes with 400at% H
			Martian Regolith w/20% Polyethylene
			Lunar Regolith w/20% Polyethylene
			Carbon Nanotubes with 200at% H
			Magnesium Borohydride w/50% Boron Nitride Nanotubes
			Boron w/30% Epoxy (Natural)
			Boron w/30% Epoxy (^10^B)

The metals evaluated were chosen as structural materials that can resist, without excessive deformation or failure, stresses that occur during launch, re-entry, deployment and service. Martian and Lunar regloith were included in this category due to their large metals components and similar densities. The fuels, hydrides and liquid gases include common fuels and propellants that will be needed on deep space missions. This category also includes the shielding materials that have been shown to have the ability to efficiently absorb the energy of the solar and cosmic radiation particles, as well as minimize the formation of secondary radiation. The Polymers and composites include the materials that have been suggested as potential materials for deep space missions [6]. This category also includes engineered materials developed to significantly increase the absorption of secondary radiation and materials in the early stages of development for the purpose of meeting both shielding and structural needs of future spacecraft missions, for example hydrogen storage in carbon and boron nitride nanotubes.

Supplementary Tables show the E_D_ absorbed in the MAX and FAX human phantoms at shielding thickness of 0.01, 0.1, 0.3, 0.5, 0.75, 1.0, 3.0, 5.0, 7.5, 10, 30, 50, 75, 100, 300, 500, 750 and 1000 g/cm^2^. Table 2 shows the E_D_ using galactic cosmic rays (GCR) boundary condition and Table 3 shows the E_D_ using the solar particle events (SPE) boundary condition.

## 2 Experimental design, materials, and methods

### 2.1 Model descriptions and geometry

OLTARIS, version 3.5, analysis package is used to evaluate this detailed radiation field. Developed by the National Aeronautics and Space Administration's (NASA) Langley Research Center to enable engineering and research related space radiation calculations. This tool allows the user to easily choose the boundary condition, shielding material and geometry, mission duration, response function and human phantom. Advanced space vehicles and structures can also be evaluated using their thickness distribution analysis tools. Each simulation used the 1002 geodesic ray distribution and was run using OLTARIS′ Ray-by-Ray method. The model geometry included a human phantom placed in the center of a hollow spherical shell. Each material is defined within OLTARIS using its composition and density. Materials compositions and densities can be requested by contacting the corresponding author. Because the energy of the GCR and SPE particles are measured in MeV and atomic bonding is in the eV range, the atomic arrangement of the material is not used in the defining of a material.

### 2.2 GCR boundary condition

Galactic cosmic rays (GCRs) are composed of fully ionized stable and meta-stable isotopes. Although they include every naturally formed element, not all elements are in high abundance. Protons account for roughly 91% of the total flux, alpha particles account for approximately 8%, and heavier particles account for less than 1% of the total flux. Even though the abundance of heavy ions is relatively low (<1%), they can contribute approximately 86% of the dose equivalent in a lightly shielded vehicle like a space suit [7]. For heavier shielded vehicles, this percentage drops off due to energetic light ions created from nuclear fragmentation of these heavy ions from interaction with spacecraft materials.

The GCR model used in this study is the Badhwar-O'Neill 2010 [8,9] model, which is based on the fitting of existing balloon and satellite measured energy spectra to accurately account for the solar modulation of each element through the heliosphere. This model determines the GCR differential energy spectrum for elements from hydrogen to nickel at any given radial distance from the sun.

The 1977 solar minimum boundary condition, with particles ranging from hydrogen to ^58^Ni, is the event used in this research. The abundances for species heavier than nickel (Z > 28) are typically four orders of magnitude less than that of ^56^Fe [10] and therefore are not included in this boundary condition. During a solar minimum, the sun's magnetic field is stable which allows for infiltration of GCRs into the solar system and increases the flux of GCRs. During a solar maximum, the chaotic magnetic fields decreases the flux of GCRs but increases SPEs.

### 2.3 SPE boundary condition

Solar particle events (SPEs) are composed of a large number of protons accelerated by the Sun's magnetic field and released as coronal mass ejections. Very large coronal mass ejections are relatively rare with less than three events occurring within an 11-year solar cycle [6]; however, SPEs are sporadic in nature. There are smaller, more frequent coronal mass ejections that occur throughout a solar cycle. With such a wide variance in magnitude, duration and composition of SPE protons, a large historical SPE was chosen as the design basis event for this study.

The SPE event used in this article was recorded by the GOES 7 spacecraft and occurred between the October 19–24, 1989 [11]. This event includes the recorded proton flux for energies greater than 10, 50 and 100 MeV and is one of the highest magnitude SPE events observed in the past 30 years. Heavier elements and electrons were also measured as part of the SPE flux, but due to their limited abundances only protons are included in this model.
